# Forecasting levels of serum 25-hydroxyvitamin D based on dietary intake, lifestyle and personal determinants in a sample of Southern Europeans

**DOI:** 10.1017/S0007114523000946

**Published:** 2023-11-28

**Authors:** Ana Valer-Martinez, Carmen Sayon-Orea, José Alfredo Martínez Hernandez, Carmen De la Fuente-Arrillaga, Javier Pérez de Rojas, Fe Barcones, Miguel Angel Martínez-González, Maira Bes-Rastrollo

**Affiliations:** 1 University of Navarra, Department of Preventive Medicine and Public Health, Pamplona, Spain; 2 Department of Family Medicine, Aragon Health Service (SALUD), Zaragoza, Spain; 3 CIBERobn, Instituto de Salud Carlos III, Madrid, Spain; 4 IdiSNa, Navarra Institute for Health Research, Pamplona, Spain; 5 Navarra Public Health Institute, Pamplona, Spain; 6 Institute IMDEA Food, Madrid, Spain; 7 University of Navarra, Department of Nutrition, Food Science and Physiology, Centre for Nutrition Research, Pamplona, Spain; 8 Department of Medicine and Psychiatry, University of Zaragoza, Instituto de Investigación Sanitaria Aragón (IIS Aragón), Zaragoza, Spain; 9 Harvard T.H. Chan School of Public Health, Boston, MA, USA

**Keywords:** Vitamin D, Predictive model, Lifestyle, Predictors

## Abstract

Vitamin D is an essential nutrient to be consumed in the habitual dietary intake, whose deficiency is associated with various disturbances. This study represents a validation of vitamin D status estimation using a semi-quantitative FFQ, together with data from additional physical activity and lifestyle questionnaires. This information was combined to forecast the serum vitamin D status. Different statistical methods were applied to estimate the vitamin D status using predictors based on diet and lifestyle. Serum vitamin D was predicted using linear regression (with leave-one-out cross-validation) and random forest models. Intraclass correlation coefficients, Lin’s agreement coefficients, Bland–Altman plots and other methods were used to assess the accuracy of the predicted *v*. observed serum values. Data were collected in Spain. A total of 220 healthy volunteers aged between 18 and 78 years were included in this study. They completed validated questionnaires and agreed to provide blood samples to measure serum 25-hydroxyvitamin D (25(OH)D) levels. The common final predictors in both models were age, sex, sunlight exposure, vitamin D dietary intake (as assessed by the FFQ), BMI, time spent walking, physical activity and skin reaction after sun exposure. The intraclass correlation coefficient for the prediction was 0·60 (95 % CI: 0·52, 0·67; *P* < 0·001) using the random forest model. The magnitude of the correlation was moderate, which means that our estimation could be useful in future epidemiological studies to establish a link between the predicted 25(OH)D values and the occurrence of several clinical outcomes in larger cohorts.

Vitamin D or cholecalciferol is a nutrient involved in a wide variety of physiological processes, including Ca and phosphorus homeostasis and bone metabolism regulation^(1,2)^. Vitamin D nuclear receptor is widely distributed among a great range of tissues in the human body, with diverse functions such as immune system and neuroendocrine system regulation and cardiometabolic control^(3)^.

In humans, the main source of vitamin D is cutaneous exposure to sun radiation, which elicits the conversion of 7-dehydrocholesterol to vitamin D in the skin^(2)^. Another source that has become essential in Northern countries, where the sunlight becomes insufficient in winter months, is vitamin D supplementation^(2)^. The dietary consumption comes habitually from natural sources (e.g. fish, eggs), fortified products (e.g. dairy products, cereals) and supplementation^(2)^. The European Food Safety Authority has defined adequate dietary values for vitamin D for all population groups^(4,5)^. Thus, for infants aged 7–11 months, a vitamin D intake of 10 mcg/d (400 μg) has been recommended. The recommended intake for older children and adults up to 70 years is 15 mcg/d (600 μg). Finally, for adults older than 70 years old, the threshold has been set at 20 mcg/d (800 μg)^(4,5)^. An irregular or inadequate intake can lead to chronic deficiency. The most common way to define vitamin D status in humans is serum levels of 25-hydroxyvitamin D (25(OH)D) determination^(6)^. However, unnecessary blood testing is not beneficial and leads to important economic costs that must be taken into account^(6)^.

Although vitamin D deficiency threshold is not clearly defined, it seems that the most accepted cut-off point may be serum concentration below 30 nmol/l (< 15 ng/ml), whereas vitamin D insufficiency includes serum concentrations between 30 and 50 nmol/l (15–20 ng/ml)^(2,5,7,8)^.

Therefore, it may be difficult to determine an accurate prevalence of vitamin D deficiency worldwide. However, some countries have described a prevalence of 60 % (Western Europe) and 36 % (the USA), among adults^(9)^. Pregnant women and new-borns seem to show the highest risk of vitamin D deficiency, as well as obese and elderly people^(10)^. Over the past decade, there has been a substantial increase of blood sample requests to determine levels of serum vitamin D, resulting in a significant cost burden on the different health care systems^(11)^. This trend may be the result of an increasing recognition of the role that vitamin D plays in musculoskeletal disorders and other physiological processes (CVD, neoplastic processes, obesity, type 2 diabetes mellitus, mental health disorders or autoimmune diseases)^(12)^. A screening strategy to help predicting serum vitamin D levels and therefore, identify those individuals that could be at risk of vitamin deficiency may be valuable, given that previous research has identified that vitamin D status is associated with different socio-demographic and lifestyle factors^(13–17)^. These predictors include vitamin D dietary intake, use of supplements, sun exposure, age, adiposity, physical activity, time spent outdoors, blood draw season and among others^(13–17)^. Several studies have developed predictive models to foretell vitamin D deficiency in adults, being most of these published articles based on populations from the USA, the Netherlands or Australia^(18–21)^. Other analyses have been conducted in specific groups such as older women with a higher risk of fracture^(22)^, pregnant women^(23,24)^ or children^(25,26)^. In addition, there is a current trend of developing predictive models based on machine learning algorithms^(27–29)^. Machine learning is an alternative statistical method that can lead to improved and steady predictions in contrast with other standard models^(27)^. However, there is not much evidence on how accurately these models predict 25(OH)D status in Mediterranean populations. Indeed, as sunlight is one of the principal sources of vitamin D, and this may vary depending on latitude, geographical location or even a different lifestyle, those predictive models may not be suitable for the Spanish population.

This study aimed to offer an alternative predictive model based on different statistical models (linear regression and machine learning models) to forecast 25(OH)D serum levels from self-reported questionnaires about dietary and lifestyle habits from a sample of Southern Europeans. In other words, this study intends to provide a good and easy to use guide, which could be used instead of blood sampling.

## Methods

### Study population

This study was conducted in two different samples of Spanish populations. The first group based on 100 subjects was collected from students and workers from the University of Navarra, whereas the second group was a sample of 120 people recruited through different health care centres from Zaragoza, all of them Caucasian. At first, 150 subjects from Zaragoza were randomly invited to join the study, from which thirty declined the invitation, whereas nearly half of the individuals who received the invitation from the University of Navarra refused to participate. The unique inclusion criteria adopted were being over the age of 18 and willingness to provide blood samples and to complete a battery of questionnaires. None of the participants had a history of cancer, severe kidney disease or pregnancy at the time of the study. Blood samples were extracted from all participants to determine their 25(OH)D levels, especially during winter and spring months, mostly in May. They also completed questionnaires including an FFQ^(30)^, leisure-time physical activity^(31)^ and other questionnaires^(32)^.

Sample size was determined according to a study based on French population^(16)^. Considering an alpha risk of 0·05, a power of 80 % and a common standard deviation of 10 in a two-sided test, the sample size was calculated to be 220. The drop-out rate was considered to be 10 %.

Diet was assessed according to a well-designed, validated and semi-quantitative FFQ^(30)^ which contains 136-item information about participants’ usual food intake in the past year. For each item, common portion sizes were specified, and consumption frequencies were divided into nine categories ranging from ‘never or almost never’ to ‘more than six servings per day’. A dietitian updated the nutrient database by using the latest available information included in the food composition tables in Spain, after processing all the questionnaires. Nutrient intake scores were calculated using an *ad hoc* computer programme, which was developed specifically for this purpose. The sum of the frequency of a given food item consumption multiplied by the nutrient composition of the specified food portion was calculated. This questionnaire also includes information about supplementation intake, sun exposure, history of sunburns and the use of solar protective measures^(30)^. Intake of vitamin D supplementation was calculated using the frequency of daily consumption of vitamin D supplementation multiplied by the amount of vitamin D of each pill derived from the brand of vitamin D supplementation or multivitamin supplement provided for each participant. Additionally, data regarding physical activity were collected through another questionnaire – also validated^(31)^. It contains information about anthropometric measures (weight, height, waist and hip perimeter), level of physical activity, time spent walking and doing up to seventeen other activities. When quantifying the amount of physical activity carried out during leisure time, each activity was weighted using its proportional number of metabolic equivalents (MET). This was done using the Compendium of Physical Activities, assigning a metabolic rate index (MET score) for each activity. Time spent on each activity (according to time per week and months per year) was multiplied by its corresponding MET score. Afterwards, all MET scores for each activity and participant were added so as to obtain a global value of MET-h/week for each volunteer. It should also be noted that participants of the study showed great variability in the length of time spent on leisure activities during weekends as a consequence of the lack of routine, which may cause an overestimation of the time spent in each activity. For this reason, all activities carried out during the weekend were disregarded, as they did not improve the statistical significance of the prediction. Information about alcohol consumption, history of chronic diseases as well as a brief questionnaire about Mediterranean diet adherence were also assessed. This study was conducted according to the guidelines laid down in the Declaration of Helsinki, and all procedures involving research study participants were approved by the Research Ethics Committee from Aragon (PI20–425). Written informed consent was obtained from all subjects.

### Laboratory analysis

Plasma 25(OH)D levels from Zaragoza’s sample were determined by electrochemiluminescence immunoassay (Elecsys® Vitamin D total II). The immunoassay employs a competitive protein-binding assay, which uses vitamin D-binding protein for detection of 25-OH Vitamin D. Serum vitamin D status from Pamplona’s sample was determined by chemiluminescent microparticle immunoassay. Both assays are intended for the quantitative determination of total 25-hydroxyvitamin D in human serum. Both methods are standardised in accordance with the Vitamin D Standardization Program. The 25-hydroxyvitamin D values ranged between 14·1 and 232 nmol/l as measured by the Reference Method, ID-LC-MS/MS (provided by the Vitamin D Standardization and Certification Program with assigned values by the RMP at CDC), showing a correlation coefficient of 0·98. The sponsor defined the limit of detection as 7·5 nmol/l (3 ng/ml) and the limit of quantitation as 12·5 nmol/l (5 ng/ml). The potential cross reactivity with 24,25(OH)D2 showed a mean of 12·4 % for non-normalised samples and 13·7 % for normalised samples. They are equivalent and no statistically significant differences have been found between them^(33)^.

### Statistical analysis

Two different statistical analyses were carried out using both statistical software STATA version 14 and R version 4.0.5. Regarding data validation of FFQ and physical activity questionnaire as serum vitamin D predictors, the relationship between the main independent variables (amount of dietary vitamin D, hours of sunbathing per week, etc.) and serum vitamin D as a continuous dependent variable was established.

A first data analysis was carried out in STATA using multiple linear regression, as a classical model. The first variable to be studied was vitamin D dietary intake. Values of vitamin D ingested from diet and vitamin supplements were joined in the same variable. This variable was adjusted for total energy intake (kcal) by the residuals method specifically for each sex, in order to avoid a possible bias. Subsequently, the following variables were included in the model: age and sex; BMI, which was calculated from self-reported weight and height (kg/m^2^); skin reaction after sun exposure (mild or severe reaction); time spent on daily walks (minutes per day) and summer sun exposure (hours per day). In order to estimate the last variable, a weighted average of the weekly sun exposure during summer was performed. Winter sun exposure was withdrawn due to the lack of statistical significance.

After adjusting linear regression models of serum vitamin D according to the variables listed above, beta coefficients were obtained and included in a predictive equation for serum vitamin D levels. Thus, successive equations were generated, each one closer to a correlation of 1 than the previous. Afterwards, a sensitivity analysis of these results was carried out through leave-one-out cross validation to obtain impartial estimations which were independent from the data that were used to derive the coefficients (training set), thus avoiding over-fitting (excessive adjustment after validating the predictive equation using the same subjects already involved to create it). This method consists in performing n linear regression models (one per participant in the study, *n* 220). Each model is applied to a sample of *n*-1 participants, where the regression coefficients for the different predictors obtained in each regression are applied only to the individual who remained outside the regression model. Thus, each subject will have a coefficient based on the predicted model for the remaining 219 participants. This method avoids over adjustment and achieves a predictive equation for each individual that comes from their own independent sample.

Another sensitivity analysis was performed using R software, where two comparable datasets called training set and testing set, which contained 110 individuals each, were randomly generated and no significant differences were found between them (*P* > 0·05). The training set was used to develop the predictive models, and the testing set was used to validate them and evaluate the root mean square error (RMSE), in order to choose the best predictive model. Several machine learning models have been tested for the purposes of this study, including random forest (RF), regression trees and linear regression models with automatic selection of predictors.

Multiple linear regression allows generating a linear model in which the value of the dependent variable (y) is determined from a set of independent variables called predictors (x_1_, x_2_, x_3_…). Predictors’ selection was carried out using *stepAIC* function. A RF model is made up of a set of individual decision trees, each trained with a random sample extracted from the original training data by bootstrapping. This implies that each tree is trained with slightly different data. In each individual tree, the observations are distributed by bifurcations (nodes) generating the structure of the tree until it reaches a terminal node^(34)^. The prediction of a new observation is obtained by adding the predictions of all the individual trees that make up the model. In addition, we get the ‘Variable Importance /Gini Index’ values for the forest, which can be used for making sense of the model but not as a multiplication factor^(34)^. Finally, regression trees are the subtype of prediction trees that are applied when the response variable is continuous. In general terms, in the training of a regression tree, the observations are distributed by bifurcations (nodes) generating the structure of the tree until it reaches a terminal node. The tree prediction is the response variable’s mean of the training observations that are at the same terminal node.

In all three models, the same strategy was applied to the training dataset, and results were repeatedly validated with cross-validation methods (K-fold cross-validation). This process consisted of dividing the data randomly into k groups of approximately the same size, k-1 groups were used to train the model and one of the groups was used as validation. This process was repeated k times using a different group as validation in each repetition. The process generated ‘k’ error’s estimations and their average was used as the final estimation. The main advantage of K-fold CV is its ability to achieve an accurate prediction of the test error, thanks to a better balance between bias and variance. Once the model was generated, it was externally validated with the dataset called test, obtaining a prediction error (RMSE) that will determine the quality of the model.

The degree of reproducibility of these models was also verified. The way to confirm it was by performing a classification by quintiles of measured and predicted 25(OH)D values and we measured the percentage of misclassification in opposite quintiles. Also, the intraclass correlation coefficient was calculated. Additionally, Bland–Altman graphs were built to assess the correlation between predicted vitamin D values and observed serum vitamin D values. Each point represents the difference between the values provided by the prediction model and the real values of vitamin D measured in serum (y axis), according to the mean between both of them (x axis). Finally, in order to assess the discrimination ability of both prediction models, we calculated the area under the receiver operating characteristic (ROC) curve (AUC), so that the models could be used as a diagnostic tool. This analysis was performed for several cut-off points due to the current debate when it comes to determining which threshold for vitamin D values is the best one to define deficiency^(35)^.

## Results

This study included a total sample of 220 healthy volunteers from Spain. The sample was composed of individuals aged between 18 and 78 years, of whom 56 % were female and 44 % male (see Table 1). The mean value of serum vitamin D was 52·8 nmol/l (sd: 19·8). Dietary intake of vitamin D showed a mean value of 8·0 (sd: 5·1) mcg/d, being fatty fish (61·7 %) the major source of vitamin D. Only sixteen individuals took vitamin D supplementation. Additionally, the parallel test and training data analysis divided the whole sample into two similar datasets with no significant differences (online Supplementary Table S1).

**Table 1.** Demographic and lifestyle characteristics of participants included in the sample (*n* 220)

Variable (units)	Mean	Standard deviation	p25	p75
Age (years)	35·1	16·7	22·0	52·0
Serum vitamin D (nmol/L)	52·8	19·8	39·5	65·0
Vitamin D from diet (mcg/d)	8·0	5·1	4·3	11·3
Vitamin D supplementation (mcg/d)*	1·82	2·24	0·33	3
Energy adjusted dietary Vit. D intake (mcg/d)†	8·1	5·2	4·3	11·3
Summer sun exposure (h/d)‡	1·2	1·6	0	2·0
BMI (kg/m^2^)§	23·2	4·1	20·4	24·7
Physical activity (MET-h/wk)	38·6	30·7	14·1	51·3
Alcohol intake (g/d)	6·6	9·3	1·7	9·1
Walking time (min/d)||	54·6	36·0	25·0	90·0
Hanging out with friends (h/d)¶	1·5	1·2	0·6	1·9
Variable	Proportion (%)			
		%		
Sex	Women	56		
	Men	44		
Skin reaction after sun exposure**	Mild reaction	74·5		
	Severe reaction	25·5		

* Only sixteen individuals took vitamin D supplementation and the values showed in the table reflect the mean intake (mcg/d) among consumers of vitamin supplements.

† Dietary vitamin D and supplementation, energy-adjusted by residual method (mcg/d).

‡ Average sun exposure time in summer during the week in the last year (h/d).

§ BMI.

|| Average walking time (min/d).

¶ Average time going out with friends during the week (h/d).

** Skin reaction in childhood or adolescence after sun exposure for at least 2 h without sun cream.

After comparing all the RMSE obtained from all the different models tested, the one that seemed to predict more accurately serum vitamin D status was the RF model, which showed the lowest RMSE of 6·79, and so it was further develop (online Supplementary Table S2). Additionally, the linear model showed an RMSE of 7·17 and 7·46 after leave one out-cross validation. The variables used to develop the linear predictive model were age, sex, vitamin D ingested from diet and supplement sources adjusted by the total energy intake, summer sun exposure, BMI, daily time spent walking, skin reaction after sun exposure and total physical activity (measured in MET-h/week). Although vitamin D intake does not show a significant correlation with serum vitamin D status, the variable was included in both models since scientific evidence describes a direct association between both of them^(5)^. The impact of alcohol consumption, time hanging out with friends, Ca intake (mcg/d) and adherence to Mediterranean diet were also studied. However, no statistically significant relationship (*P* > 0·05) was observed in relation to vitamin D values. The estimated equation for the linear prediction model has been included in Table 2. After leave-one-out cross-validation, coefficients remain similar (online Supplementary Table S3). RF model included slightly different variables: age, sex, total amount of vitamin D ingested and adjusted for the total energy intake (kcal), BMI (kg/m^2^), summer sun exposure (h/d), daily time spent walking and hanging out with friends (h/d), skin reaction after sun exposure, alcohol consumption (g/d) and physical activity (MET-h/week) (online Supplementary Fig. S1). Ca intake (mcg/d) and adherence to Mediterranean diet were also studied, but no significant improvement was seen in the model. Therefore, these two last variables were withdrawn to simplify the prediction.

**Table 2.** Detailed stages included in the development of the multiple linear regression model

Steps	Variables	Linear regression model equation	Pearson correlation coefficient between linear prediction and serum vitamin D	95 % CI
1	Energy adjusted vitamin D intake	Linear prediction 1 = 19·33 + (0·20 × vitd_a)*	0·13	0·001, 0·26
2	Age and sex	Linear prediction 2 = 21·15 + (0·13 × vitd_a) + (–0·06 × age) + (1·67 × sex)	0·22	0·09, 0·34
3	BMI	Linear prediction 3 = 29·57 + (0·11 × vitd_a) + (–0·002 × age) + (2·06 × sex) + (–0·44 × bmi)†	0·30	0·17, 0·41
4	Skin reaction	Linear prediction 4 = 30·92 + (0·09 × vitd_a) + (–0·02 × age) + (1·83 × sex) + (–0·43 × bmi) + (–2·99 × skin)‡	0·34	0·22, 0·45
5	Walking	Linear prediction 5 = 27·90 + (0·06 × vitd_a) + (–0·04 × age) + (1·72 × sex) + (–0·36 × bmi) + (–3·27 × skin) + (0·04 × walk_min)§	0·37	0·25, 0·48
6	Summer sun exposure	Linear prediction 6 = 26·16 + (0·06 × vitd_a) + (–0·02 × age) + (1·29 × sex) + (–0·33 × bmi) + (–3·61 × skin) + (0·04 × walk_min) + (0·80 × sun)||	0·41	0·30, 0·52
7	Physical activity	Linear prediction 7 = 24·4 + (0·04 × vitd_a) + (–0·01 × age) + (1·37 × sex) + (–0·31 × bmi) +(–3·71 × skin) + (0·03 × walk_min) + (0·77 × sun) + (0·03 × phys_act)¶	0·43	0·31, 0·53

* vitd_a: Dietary vitamin D and supplementation, energy-adjusted by residual method (mcg/d).

† BMI (kg/m^2^).

‡ skin: skin reaction after sun exposure (0: mild; 1: severe).

§ walk_min: walking time (min/d).

|| sun: sun exposure during summer (h/d).

¶ phys_act: physical activity measured by metabolic equivalents (MET-h/week).

We classified measured and predicted 25(OH)D values by quintiles, from which only 3·2 % were placed in opposite quintiles by the linear model, whereas 0·9 % of the values were misclassified in opposite quintiles by RF’s model (online Supplementary Table S4).

The intraclass correlation coefficient was 0·60 (95 % CI: 0·52, 0·67, *P* < 0·001) for the RF’s model and 0·31 (95 % CI: 0·18, 0·42, *P* < 0·001) for the linear prediction models.

**Fig. 1.**
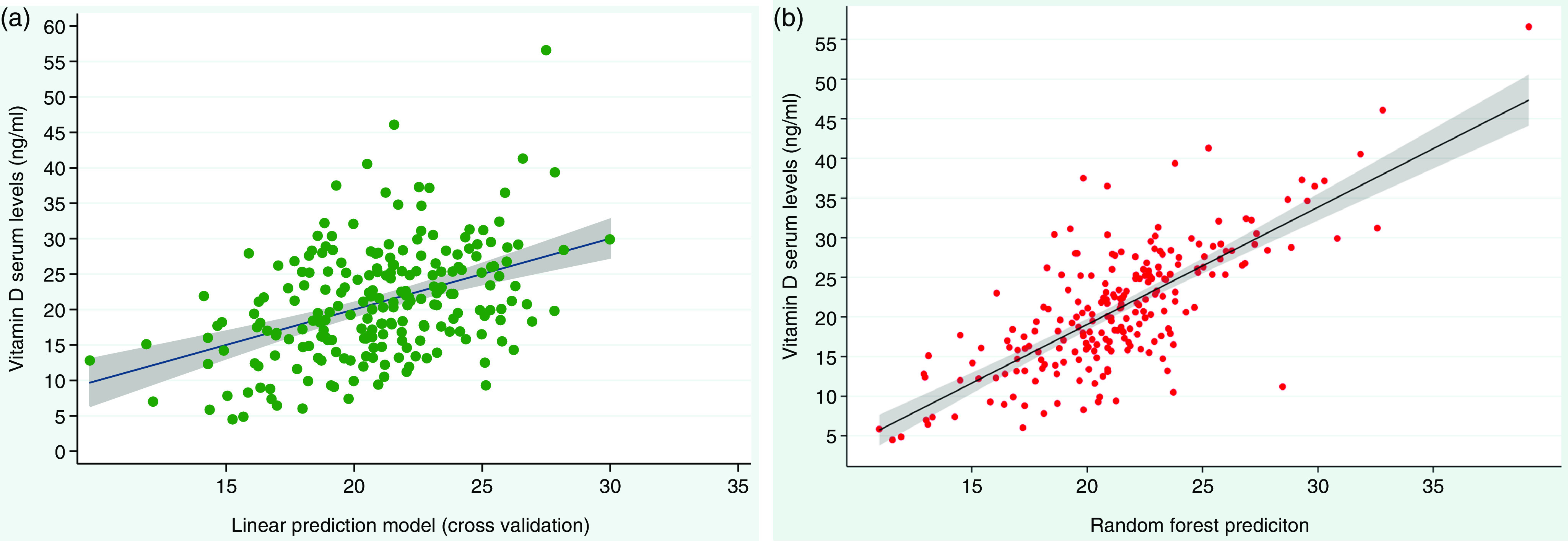
Linear correlation graphs between vitamin D serum levels and predictive values for the linear prediction model after leave-one-out cross-validation (a) and random forest (b). *Linear correlation graph after leave-one-out cross-validation (LOOCV) minimally differs from the linear correlation graph before LOOCV.

**Fig. 2.**
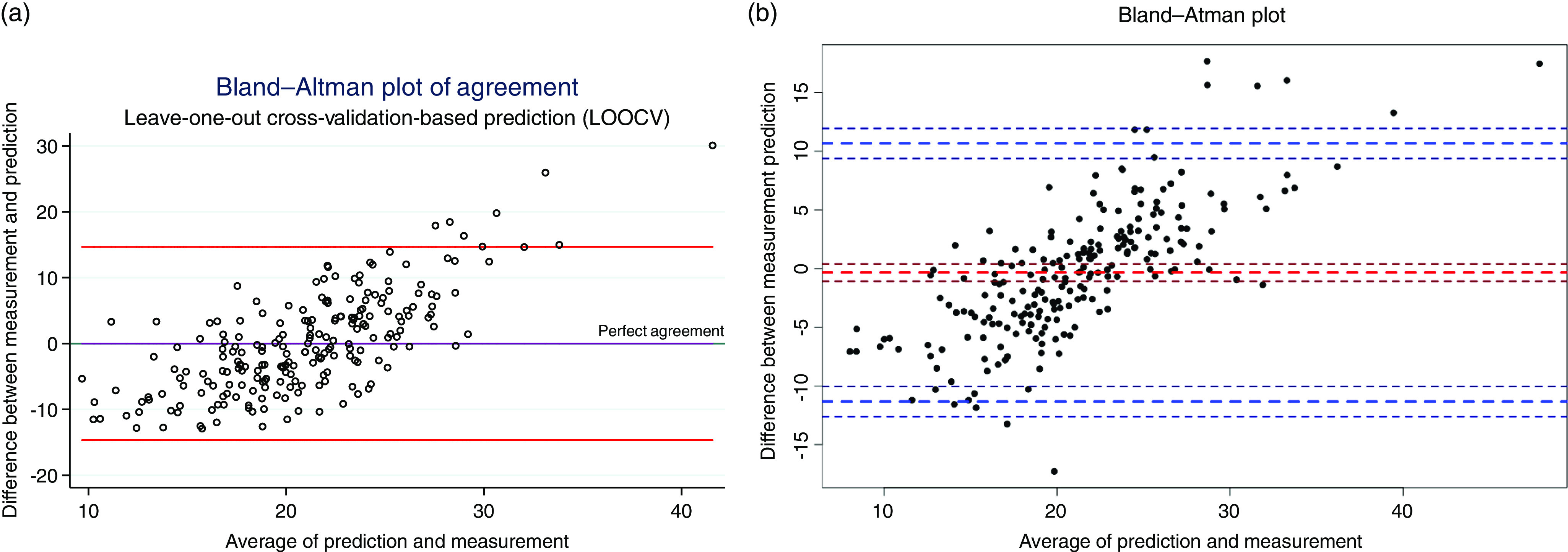
Bland–Altman plots of predicted vitamin D from multivariable linear regression model after cross-validation (a) and from random forest model (b). The sidelines indicate 95 % of agreement, whereas the middle line indicates the perfect agreement. *Linear correlation’s Bland–Altman graph after leave-one-out cross-validation (LOOCV) minimally differs from the linear correlation graph before LOOCV.

Figure 1 shows two graphs, the linear correlation graph from the linear prediction models involving the whole sample after adjustment by the leave-one-out cross-validation. The second graph shows the correlation between vitamin D serum values and RF model prediction. In addition, Bland–Altman graphs (see Fig. 2) show that most of the points (at least 95 %) lie between the tolerance limits. Lin’s concordance correlation coefficient, which measures absolute agreement, was found to be 0·60 (95 % CI: 0·54, 0·65, *P* < 0·001) for RF’s prediction and 0·31 (95 % CI: 0·22, 0·39, *P* < 0·001) for the linear prediction validated model.

**Fig. 3.**
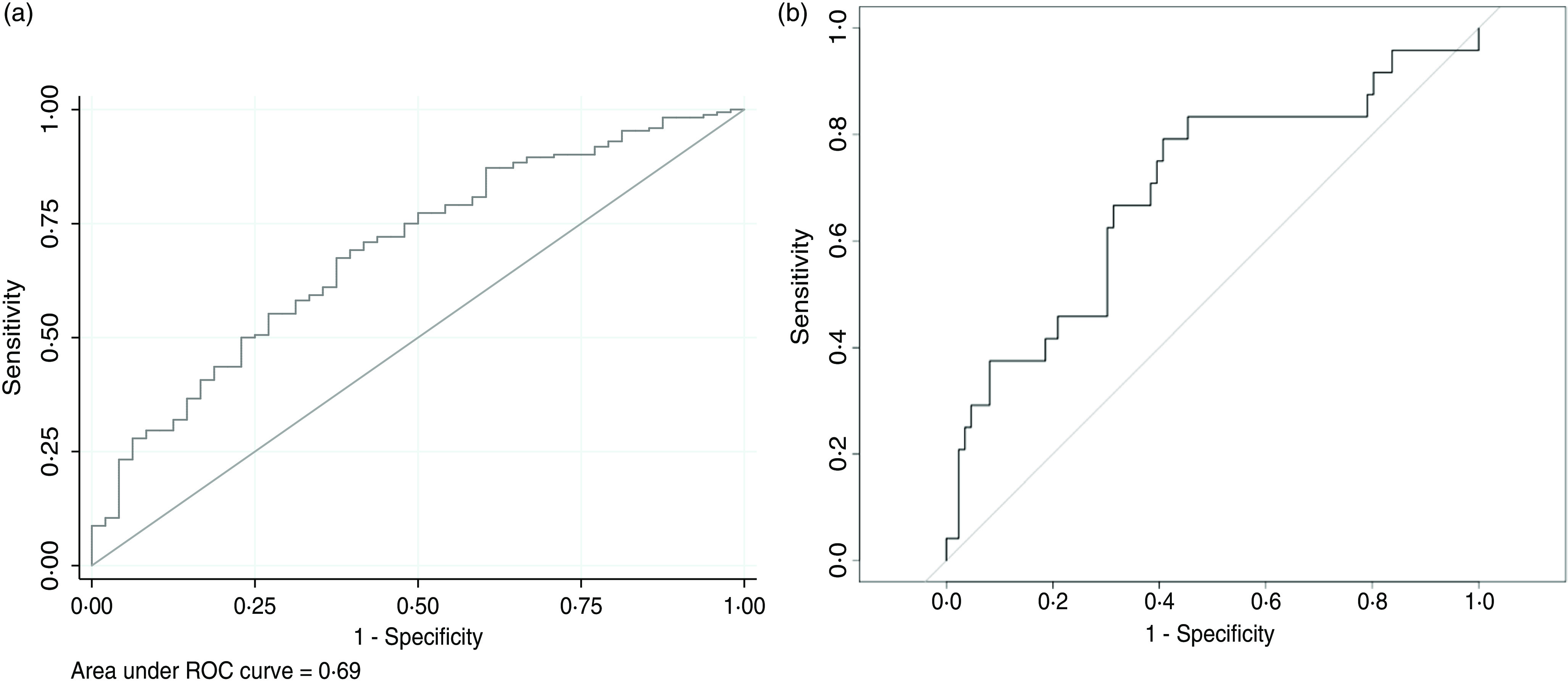
Linear model and random forest ROC curves for a cut-off point of 37·5 nmol/l. (a) Linear model’s area under ROC curve = 0·69 (95 % CI 0·59, 0·77; *P* < 0·001); (b) Random forest’s area under ROC curve = 0·69 (95 % CI 0·57, 0·82; *P* < 0·001).

Finally, the AUC was also calculated for several cut-off points. If vitamin D values below 37·5 nmol/l (15 ng/ml) were considered deficient, the AUC would be 0·69 (95 % CI 0·59, 0·77; *P* < 0·001) for the linear prediction model and 0·69 (95 % CI 0·57, 0·82; *P* < 0·001) for RF’s prediction (Fig. 3). Considering the lower limit 50 nmol/l (20 ng/ml), the linear model showed an AUC = 0·65 and the RF model AUC’s was 0·67. When a threshold of 62·5 nmol/l (25 ng/ml) was set, AUC were 0·62 and 0·67, respectively.

## Discussion

This study aimed to build an approach concerning a predictive model of serum vitamin D using seven different variables from a self-reported questionnaire based on two different statistical approaches, multiple linear regression and RF.

The magnitudes of the correlation found in this study were moderate for the multiple linear regression model and strong for RF’s prediction model. In other words, after making adjustments to include several variables, it may be possible to estimate vitamin D levels in epidemiological studies, and therefore prospectively study the long-term impact on health in similar cohorts. These findings agree with previously published literature in which different machine learning models, including RF, seemed to perform better prediction due to a better goodness-of-fit^(27,28)^. What is more, RF model has been proposed as a more appropriate model to deal with small sample size or ‘large p value’ issues, in comparison with other models such as linear regression^(34)^.

Another noticeable finding was the low association between the values of vitamin D ingested from diet and the values measured in blood, which could be explained by the sample’s low dietary intake levels and the type of population from which the sample was taken. The USA National Academy of Medicine recommends a daily vitamin D intake of 15 mcg for children and adults up to 70 years of age and 20 mcg for adults over 70^(5)^. However, the mean intake of vitamin D intake in this study (including diet and supplementation) was 8·10 ± 5·17 mcg/d.

Relationship between alcohol intake and serum 25(OH)D remains unclear as no significance correlation was found in the linear regression model, although RF’s prediction model showed a relevant association between the two variables. A systematic review, which examined such association, reported conflicting findings^(36)^. Thus, a positive association was reported in fifteen studies, whereas an inverse association was described in eighteen papers and no association in another sixteen publications. However, the authors concluded that most of the studies, which observed a positive association, were more recent and showed more consistent methodology, than the others^(36,37)^. Another study found that vitamin D deficiency was associated with no alcohol consumption in the past 12 months^(21)^. The authors concluded that as alcohol consumption was associated with greater social interaction and consequently, higher sunlight exposure, people who consume alcohol may be less likely to show insufficient levels of serum 25(OH)D^(21)^.

In addition, Bland—Altman graphs were important to evaluate the differences at several magnitudes of the observed and predicted vitamin D. These differences might allow us to investigate any possible relationship between measurement error and the true value. Despite the fact that most of the dots are included between the limits of agreement, the graphs show a proportional variability, there is a slight negative bias across lower ranges of vitamin D, whereas at higher 25(OH)D concentrations there is a tendency towards positive bias, even a slight dispersion. Moreover, there is a rather large range between the limits of agreement, which may affect the estimation’s accuracy. For these reasons, sensitivity analysis such as the exclusion of outliers and the comparison between extreme quintiles should be taken into consideration in future studies when vitamin D prediction will be used.

Our model showed that the best predictive cut-off point for vitamin D was 37·5 nmol/l (15 ng/ml), which showed an AUC of 0·69. This means that 69 % of the participants were properly classified. Our findings are in agreement with the rest of those previously carried out on the validation of diet and exercise questionnaires for predicting vitamin D values. The highest sensitivity’s values were described by Annweiler *et al.* ^(38)^ who observed 98 % (AUC = 0·93) and 87 % (AUC = 0·86) to identify higher cut-off points of serum 25(OH)D, < 75 nmol/l and 50 nmol/l, respectively. Cut-off points below 25 nmol/l showed a sensitivity of 64·9 % and an area under the curve of 0·38. Deschasaux *et al.* ^(16)^ found an area under the curve of 0·70 after considering values below 20 ng/ml as threshold for vitamin D deficiency. In this context, Sohl *et al.* ^(19)^ developed a model to predict values of serum 25(OH)D below 50 nmol/l and 30 nmol/l and reported areas under the curve of 0·71 and 0·80, respectively. Moreover, Tran *et al.* ^(20)^ developed a model with two cut-off points of serum 25(OH)D < 25 nmol/l and < 50 nmol/l for Australian older adults and found an area under the curve of 0·82 and 0·73, respectively. Another study carried out in New Zealand showed an AUC of 0·72 in the detection of serum 25(OH)D < 40 nmol/l^(21)^. Other populations were also studied, such as Japanese adults^(39)^, where a sensitivity of 61 % and specificity of 79 % (AUC = 0·75) were able to detect values of serum vitamin D under 50 nmol/l, and a Brazilian older community^(40)^, where an AUC of 0·68 was found. An advantage of the present study (compared with previous ones) is the application of more appropriate methods to estimate the prediction’s validity, such as the intraclass correlation coefficient, the Lin concordance coefficient or the Bland–Altman graph. Jensen *et al.* ^(23)^ also calculated a Cohen’s weighted kappa coefficient of 0·3. They classified individuals by quintiles of measured and predicted 25(OH)D values, from which 69·9 % were placed in the same or adjacent quintile and only 1·9 % were placed in opposite quintiles. Another study cross-classified measured and predicted 25(OH)D status and found that 59·8 %–66·5 % were placed in the same or adjacent quintile, concluding that their model could be used to rank individuals by their vitamin D status^(18)^.

Among the limitations of this study, it is important to note that the number of participants was limited and the sample homogeneous to assess any clear correlation for variables such as ethnicity and skin colour with vitamin D values. Therefore, this model is likely to be generalisable only to similar healthy cohorts, although approaches could be taken to develop similar models in other populations. Furthermore, the sample included healthy adults with a sufficient mean of serum vitamin D status, leading to a selection bias, which determines that the model is better detecting individuals with sufficient levels of vitamin D than insufficiency. However, this fact has not influenced the selection of vitamin D determinants, as they have proved to be similar to other cohorts with lower levels of serum vitamin D^(20,39)^. In this line, the results obtained may not provide a precise identification of the individuals at higher risk and may not be suitable for particular groups such as pregnant women and children, in which specific prediction models should be used to reach adequate outcomes, as they show different metabolic and physiological processes. In this sense, this study may be more useful in the epidemiological and research fields, rather than the clinical setting. In addition, the answers to questionnaires were self-reported by participants; so, such answers could suffer from classification bias, although in this case it would be a non-differential bias, because the participants did not know their own vitamin D levels at the time they filled the questionnaires. Another important fact is that a single blood measure cannot be considered as a true gold standard of long-term average 25(OH)D concentration. Furthermore, an important amount of the blood samples were obtained in spring. This fact could have influenced serum vitamin D values, as winter sun exposure seemed to be lower than in summer, observing as a result lower values of serum vitamin D than the average (median of 51·38 nmol/l). In addition, there are some variables that may play an important role in estimating vitamin D levels, such as latitude of residence or type of clothing, which were not analysed.

A strong point of our study is the inclusion of different predictive models and their comparison. Most of the published studies have developed models based on linear regression, especially logistic regression. However, prediction of vitamin D made by RF and other machine learning models have proved to be effective. Another positive impact our study may bring is avoiding unnecessary blood tests and vitamin supplementation, neither of them risk-free practices. Although blood sampling of 25(OH)D is the most accurate way of measuring serum vitamin D, our methods provide a rapid, computer-based and not expensive tool to include subjects in future studies.

### Conclusion

The model that best predicted serum vitamin D levels was the RF model, which showed a magnitude of the correlation that can be rated as strong, in comparison to the MLR model, which showed a moderate correlation. For this reason, both predictive models could be useful in future epidemiological studies to assess the risk of suffering from different diseases within a larger cohort. This approach is justified when we consider that there is recent evidence that vitamin D does play a significant role in several physiopathological processes, not only in Ca homeostasis but also in the regulation of both the immune and inflammatory systems, as well as in neuroendocrine and cardiovascular functions. Furthermore, the use of predictive models such as the current could avoid invasive procedures, such as unnecessary blood tests, as well as the overprescription of vitamin supplements.

## Supplementary material

For supplementary material accompanying this paper visit https://doi.org/10.1017/S0007114523000946. click here to view supplementary material
