# Supplementary material for: Forecasting levels of serum 25-hydroxyvitamin D based on dietary intake, lifestyle and personal determinants in a sample of Southern Europeans
Source: Br J Nutr. 2023 Apr 11;130(10):1814–22. doi: 10.1017/S0007114523000946 (PMC10587381; doi:10.1017/S0007114523000946)
Supplement: Supplementary file 1 [file S0007114523000946sup001.zip › S0007114523000946supp001.docx]

Table S2. Prediction performance of the test dataset (RMSE)

| Linear Model (STATA^®^) | Linear Model after LOOCV (STATA^®^) | Linear model glmStepAIC | Random Forest | Decision Tree |
| --- | --- | --- | --- | --- |
| 7.17 | 7.46 | 7.46 | 6.79 | 7.51 |
