# Supplementary material for: Forecasting levels of serum 25-hydroxyvitamin D based on dietary intake, lifestyle and personal determinants in a sample of Southern Europeans
Source: Br J Nutr. 2023 Apr 11;130(10):1814–22. doi: 10.1017/S0007114523000946 (PMC10587381; doi:10.1017/S0007114523000946)
Supplement: Supplementary file 1 [file S0007114523000946sup001.zip › S0007114523000946supp002.docx]

Table S4. Misclassification (number of participants) observed in the linear prediction model after leave-one-out cross-validation (Table A) and Random Forest model (Table B). The sample is stratified into quintiles.

| A) | | Serum vitamin D quintiles | | | | |
| --- | --- | --- | --- | --- | --- | --- |
|  |  | 1 | 2 | 3 | 4 | 5 |
| Linear prediction quintiles | 1 | 19 | 11 | 5 | 5 | 4 |
|  | 2 | 9 | 11 | 7 | 7 | 10 |
|  | 3 | 7 | 7 | 9 | 13 | 8 |
|  | 4 | 7 | 10 | 10 | 8 | 9 |
|  | 5 | 3 | 5 | 12 | 11 | 13 |

Table A shows the number of participants misclassified after comparing validated linear prediction and serum vitamin D quintiles. Percentage of misclassification in opposite quintiles was 3.2%

| B) | | Serum vitamin D quintiles | | | | |
| --- | --- | --- | --- | --- | --- | --- |
|  |  | 1 | 2 | 3 | 4 | 5 |
| Random Forest’s prediction quintiles | 1 | 24 | 11 | 5 | 1 | 2 |
|  | 2 | 12 | 14 | 10 | 7 | 1 |
|  | 3 | 4 | 10 | 16 | 10 | 3 |
|  | 4 | 2 | 4 | 8 | 19 | 11 |
|  | 5 | 0 | 5 | 5 | 7 | 27 |

Table B shows the number of participants misclassified after comparing Random Forest’s prediction and serum vitamin D quintiles. The percentage of misclassification in opposite quintiles was 0.9%
