# Supplementary material for: Forecasting levels of serum 25-hydroxyvitamin D based on dietary intake, lifestyle and personal determinants in a sample of Southern Europeans
Source: Br J Nutr. 2023 Apr 11;130(10):1814–22. doi: 10.1017/S0007114523000946 (PMC10587381; doi:10.1017/S0007114523000946)
Supplement: Supplementary file 1 [file S0007114523000946sup001.zip › S0007114523000946supp003.pdf]

Figure S1. Variable importance for Random Forest model.

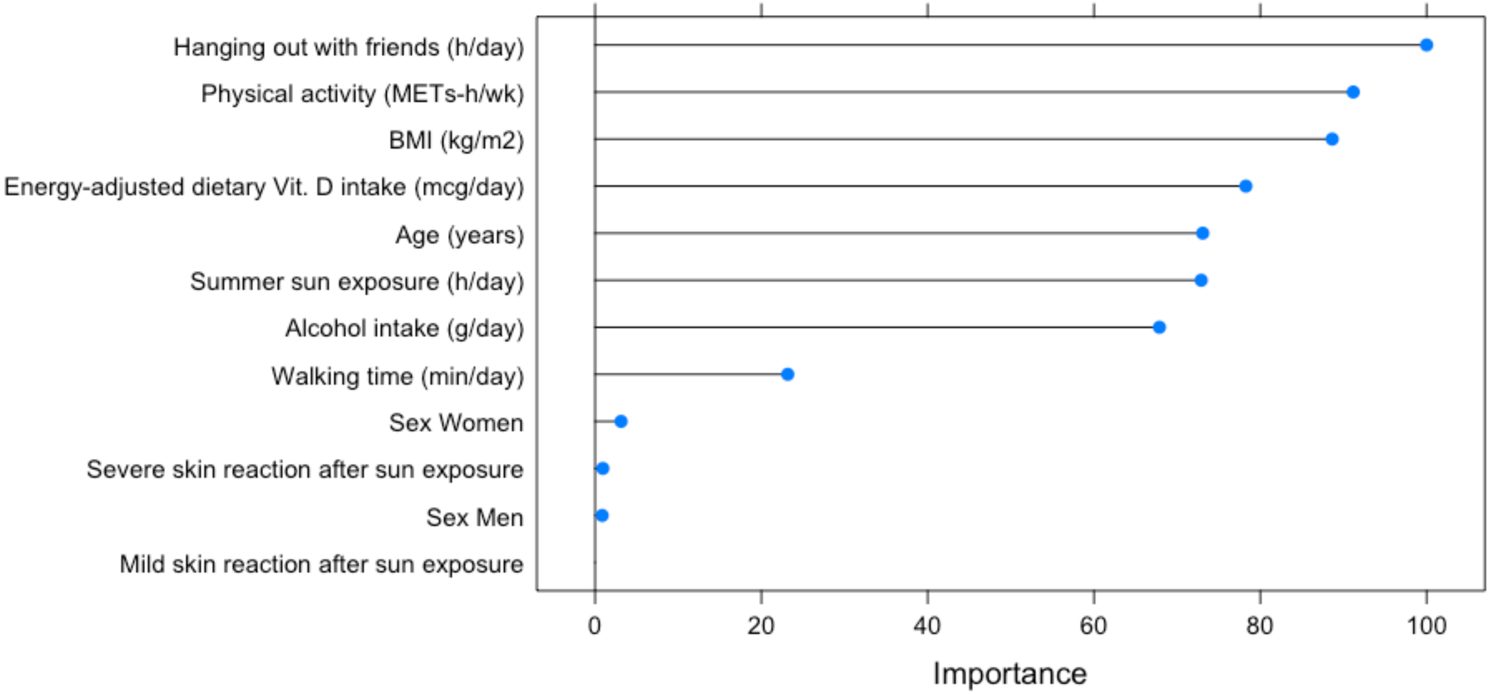

The graph shows the importance values of each variable included in the model. Each value has been weighted on the maximum importance value, which is 100%.
