# Supplementary material for: Forecasting levels of serum 25-hydroxyvitamin D based on dietary intake, lifestyle and personal determinants in a sample of Southern Europeans
Source: Br J Nutr. 2023 Apr 11;130(10):1814–22. doi: 10.1017/S0007114523000946 (PMC10587381; doi:10.1017/S0007114523000946)
Supplement: Supplementary file 1 [file S0007114523000946sup001.zip › S0007114523000946supp004.docx]

Table S1. Summary descriptive table by groups of ‘set’ represented by median (p25;p75)

| Variable (units) | All  N=220 | Test  N=110 | Training  N=110 | p overall |
| --- | --- | --- | --- | --- |
| Age (years) | 26.0 (22.0;52.0) | 25.0 (22.0; 46.8) | 26.5 (23.0;55.8) | 0.167 |
| Serum Vitamin D (nmol/L) | 51.5 (39.5;65.0) | 51.3 (39.5;64.8) | 51.5 (39.8;64.8) | 0.751 |
| Energy-adjusted dietary Vit. D intake (mcg/day) ^a^ | 6.6 (4.31;11.3) | 7.0 (4.4;11.9) | 5.9 (4.3;11.0) | 0.453 |
| Calcium intake (mcg/day) | 1258 (957;1573) | 1248 (960;1576) | 1271 (958;1566) | 0.896 |
| Summer sun exposure (h/d) ^b^ | 0.9 (0.3;2.0) | 0.9 (0.3;2.0) | 0.9 (0.3;2.0) | 0.755 |
| BMI (kg/m^2^) ^c^ | 22.2 (20.4;24.7) | 22.1 (20.5;24.7) | 22.4 (20.3;24.6) | 0.893 |
| Physical activity (METs-h/wk) | 33.5 (14.3;51.3) | 34.2 (13.6;54.7) | 32.9 (16.6;47.3) | 0.913 |
| Alcohol intake (g/day) | 4.0 (1.7;9.1) | 3.8 (1.8;8.5) | 4.6 (1.1;9.7) | 0.473 |
| Walking time (min/day) ^d^ | 45.0 (25.0;90.0) | 45.0 (25.0;90.0) | 45.0 (25.0;90.0) | 0.584 |
| Hanging out with friends (h/day) ^e^ | 1.4 (1.0;2.0) | 1.4 (1.1;2.0) | 1.4 (1.0;2.0) | 0.445 |
| Sex:  Men | 97 (44.1%) | 48 (43.6%) | 49 (44.5%) | 1.000 |
| Women | 123 (55.9%) | 62 (56.4%) | 61 (55.5%) |  |
|  |  |  |  |  |
| Skin reaction after sun exposure ^f^ |  |  |  | 0.877 |
| Mild reaction | 164 (74.5%) | 81 (73.6%) | 83 (75.5%) |  |
| Severe reaction | 56 (25.5%) | 29 (26.4%) | 27 (24.5%) |  |

(a) Dietary vitamin D and supplementation, energy-adjusted by residual method (mcg/day), b) Average sun exposure time in summer during the week in the last year (hours/day), (c) BMI: Body Mass Index, (d) Average walking time (minutes/day), (e) Average time going out with friends during the week (hours/day), (f) Skin reaction in childhood or adolescence after sun exposure for at least 2 hours without sun cream.
