# Supplementary material for: Forecasting levels of serum 25-hydroxyvitamin D based on dietary intake, lifestyle and personal determinants in a sample of Southern Europeans
Source: Br J Nutr. 2023 Apr 11;130(10):1814–22. doi: 10.1017/S0007114523000946 (PMC10587381; doi:10.1017/S0007114523000946)
Supplement: Supplementary file 1 [file S0007114523000946sup001.zip › S0007114523000946supp005.docx]

Table S3. Leave-one-out cross-validation. Median and p25-p75 from each coefficient included in the model after 220 repetitions in the multiple linear regression.

|  | Median | (P25;P75) |
| --- | --- | --- |
| Intercept | 24.40 | (24.32;24.48) |
| Coefficient (b_1_) of vitamin D intake | 0.04 | (0.04;0.04) |
| Coefficient (b_2_) of age | -0.01 | (-0.01;-0.01) |
| Coefficient (b_3_) of sex | 1.37 | (1.33;1.41) |
| Coefficient (b_4_) of body mass index (BMI) | -0.31 | (-0.31;-0.30) |
| Coefficient (b_5_) of skin phototype | -3.71 | (-3.74;-3.68) |
| Coefficient (b_6_) of walking | 0.03 | (0.03;0.03) |
| Coefficient (b_7_) of summer sun exposure | 0.77 | (0.76;0.77) |
| Coefficient (b_8_) of physical activity | 0.03 | (0.03;0.03) |
